# Development and characterization of EST‐SSR markers for *Carex angustisquama* (Cyperaceae), an extremophyte in solfatara fields

**DOI:** 10.1002/aps3.1185

**Published:** 2018-10-19

**Authors:** Koki Nagasawa, Hiroaki Setoguchi, Masayuki Maki, Hayato Goto, Keitaro Fukushima, Yuji Isagi, Shota Sakaguchi

**Affiliations:** ^1^ Faculty of Integrated Human Studies Kyoto University Yoshida‐nihonmatsu‐cho, Sakyo‐ku Kyoto 606‐8501 Japan; ^2^ Graduate School of Human and Environmental Studies Kyoto University Yoshida‐nihonmatsu‐cho, Sakyo‐ku Kyoto 606‐8501 Japan; ^3^ Botanical Gardens Tohoku University Kawauchi Sendai 980‐0862 Japan; ^4^ Graduate School of Life Sciences Tohoku University Kawauchi Sendai 980‐0862 Japan; ^5^ The Center for Ecological Research Kyoto University Otsu Shiga 520‐2113 Japan; ^6^ Graduate School of Agriculture Kyoto University Kyoto 6068502 Japan

**Keywords:** *Carex angustisquama*, *Carex doenitzii*, Cyperaceae, expressed sequence tag–simple sequence repeat (EST‐SSR) markers, solfatara

## Abstract

**Premise of the Study:**

Expressed sequence tag–simple sequence repeat (EST‐SSR) markers were developed for *Carex angustisquama* (Cyperaceae) to investigate the evolutionary history of this plant that is endemic to solfatara fields in northern Japan.

**Methods and Results:**

Using RNA‐Seq data generated by the Illumina HiSeq 2000, 20 EST‐SSR markers were developed. Polymorphisms were assessed in *C. angustisquama* and the closely related species *C. doenitzii* and *C. podogyna*. In *C. angustisquama*, many loci were monomorphic within populations; the average number of alleles ranged from one to five, and levels of expected heterozygosity ranged from 0.000 to 0.580, while all markers were polymorphic in a population of *C. doenitzii*. This indicates that low genetic polymorphism of *C. angustisquama* is likely due to the species’ population dynamics, rather than to null alleles at the developed markers.

**Conclusions:**

These markers will be used to assess genetic diversity and structure and to investigate evolutionary history in future studies of *C. angustisquama* and related species.

*Carex* L. is one of the largest and most widespread genera of the flowering plants, with approximately 2000 species (Reznicek, 1990). Most of its species are distributed in the Northern Hemisphere, especially in northern temperate and arctic regions. In addition to its global distribution, it is noteworthy that the species in the genus occur in various habitats ranging from rainforests and dry grassland to wet meadows, temperate forests, and alpine zones (Starr et al., 1999), which makes them useful models to study plant adaptation to the environment.

*Carex angustisquama* Franch. (Cyperaceae) is a perennial sedge that is endemic to solfatara fields in the Tohoku region of northern Japan. Solfatara fields are areas around fumaroles emitting sulfide gases containing H_2_S and SO_2_ even after eruption (Tsujimura, 1979; Yamamoto et al., 2018). Acidified by sulfide gases from fumaroles, the soil in solfatara fields has low pH values of 2–3 and high concentrations of sulfur and aluminum, making a harsh environment for plants to survive. *Carex angustisquama* grows close to fumaroles where no other vascular plants are able to survive (Tsujimura, 1982). Because no other closely related species in *Carex* sect. *Podogynae* Holm grow in a similar habitat, *C. angustisquama* is assumed to have adapted to solfatara fields in the process of speciation. *Carex angustisquama* also represents a disjunct geographic distribution in six main volcanic areas in the Tohoku region, which are isolated by unsuitable forested vegetation. This pattern of distribution provides an ideal model to reconstruct the historical biogeography of *C. angustisquama*.

To investigate the genetic structure and evolutionary history of *C. angustisquama*, genetic markers are needed, but there are no available markers that can be applied to this species. Expressed sequence tag–simple sequence repeat (EST‐SSR) markers are widely distributed both in transcribed and nontranscribed regions (Morgante et al., 2002). EST‐SSR markers are regarded as easier and less expensive markers to develop and reported to be more transferable among closely related species (Bouck and Vision, 2007). Moreover, they are shown to be more reliable because they have lower frequencies of null alleles than anonymous genomic SSR markers (Ellis and Burke, 2007). Therefore, we developed EST‐SSR markers and examined their polymorphisms and transferability to closely related taxa.

## METHODS AND RESULTS

Total RNA was extracted from *C. angustisquama* (population CA18, Appendix 1) using the Agilent Plant RNA Isolation Mini Kit (Agilent Technologies, Santa Clara, California, USA). A non‐normalized cDNA library was constructed and sequenced using the Illumina HiSeq 2000 system (Illumina, San Diego, California, USA). De novo assembly of 83,484,902 cleaned 100‐bp reads (DNA Data Bank of Japan [DDBJ], Bioproject PRJDB6849) using CLC Genomic Workbench version 10.1.1 software (CLC bio, Aarhus, Denmark) produced 53,628 contigs (N50: 1321 bp).

Microsatellite regions (≥8 dinucleotide repeats, ≥8 trinucleotide repeats) were searched using MSATCOMMANDER (Faircloth, 2008). We obtained 937 markers, of which 63 pairs were selected based on repeat number. For all loci, the forward primer was synthesized with one of four different M13 sequences (5′‐CACGACGTTGTAAAACGAC‐3′, 5′‐TGTGGAAT‐TGTGAGCGG‐3′, 5′‐CTATAGGGCACGCGTGGT‐3′, or 5′‐CGGA‐GAGCCGAGAGGTG‐3′) and the reverse primer was tagged with a PIG‐tail (5′‐GTTTCTT‐3′). PCR reactions were performed using a QIAGEN Multiplex PCR Kit (QIAGEN, Hilden, Germany) in a 10‐μL volume containing 20–30 ng of DNA, 5 μL of 2× Multiplex PCR Master Mix, 0.01 μM of forward primer, 0.2 μM of reverse primer, and 0.1 μM of fluorescently labeled M13 primer. The PCR protocol was as follows: 95°C for 30 min; followed by 35 cycles of 94°C for 30 s, 60°C for 3 min, 72°C for 1 min; and a final extension at 68°C for 30 min. Amplified products were loaded onto an ABI 3130 autosequencer (Applied Biosystems, Foster City, California, USA) using the GeneScan 600 LIZ Size Standard (Applied Biosystems), POP7 polymer (Applied Biosystems), and 36‐cm capillary array. Fragment size was determined using GeneMapper (Applied Biosystems).

For the initial PCR amplification trial, we used two individuals from population CA09 (Appendix 1). For the 32 primer pairs that showed clear peaks, two individuals from each population (CA09, CA13, CA14, and CA15; Appendix 1) were then used to check polymorphisms among populations. Using 20 primers that were polymorphic over the eight samples (details for 12 monomorphic markers are provided in Appendix 2), 24 individuals from each population (CA09, CA14, and CA15) were evaluated for within‐population polymorphisms. However, because few polymorphisms were detected within each population, we examined the transferability and evaluated polymorphisms in two closely related species (*C. doenitzii* Boeckeler and *C. podogyna* Franch. & Sav.; Appendix 1) to test whether low genetic variation of *C. angustisquama* was the result of null alleles at the markers or of the species’ genetic nature. GenAlEx 6.5 software (Peakall and Smouse, 2012) was used to calculate genetic diversity indices (number of alleles [*A*], observed heterozygosity [*H*_o_], and expected heterozygosity [*H*_e_]). FSTAT 2.9.3 software (Goudet, 1995) was used to test significance of Hardy–Weinberg equilibrium (HWE) by 1000 randomizations; the significance of the associated *P* values was adjusted by applying sequential Bonferroni correction. The test for the presence of null alleles was performed using MICRO‐CHECKER version 2.2.3 (van Oosterhout et al., 2004).

For *C. angustisquama*, all primer pairs (Table 1) were polymorphic when all populations were combined; *A* ranged from two to seven, and levels of *H*_e_ and *H*_o_ ranged from 0.100 to 0.703 and 0.000 to 0.286, respectively (Table 2). For each population, *A* ranged from one to five, and levels of *H*_e_ and *H*_o_ ranged from 0.000 to 0.580 and 0.000 to 0.524, respectively (Table 2). For cross‐species amplification, 20 and nine primer pairs were polymorphic in *C. doenitzii* and *C. podogyna*, respectively (Table 3). Significant departures (*P* < 0.01) from HWE were detected in three loci (Cang4398, Cang7240, and Cang48335) in *C. doenitzii*, although no significant departures were detected for any of the populations or loci in both *C. angustisquama* and *C. podogyna*. Analysis with MICRO‐CHECKER (at the 99% confidence level) highlighted the existence of null alleles at some loci in *C. angustisquama* and *C. doenitzii* (Tables 2, 3).

**Table 1:** Twenty polymorphic EST‐SSR markers developed for *Carex angustisquama*

Locus	Primer sequences (5′–3′)	Repeat motif	Allele size range (bp)	BLASTX top hit description	*E*‐value	GenBank accession no.
Cang_681	F: TGTGGAATTGTGAGCGGAGCTTATTGGCCGCATGAAC	(AG)_19_	245–251	B‐box zinc finger protein 22 [*Ananas comosus*]	8.00E‐77	FX986011
	R: GTTTCTTCCAACCGGATAAAGCTGCG					
Cang_1267	F: TGTGGAATTGTGAGCGGTAATGTGGGTCCCGGTACTG	(AGC)_9_	209–221	PREDICTED: ATP‐dependent helicase BRM [*Oryza brachyantha*]	0.0	FX985999
	R: GTTTCTTCGTGAAACCGAAACCTGGTC					
Cang_1881	F: TGTGGAATTGTGAGCGGTGTGGATGACGTGGCATTTG	(AT)_11_	316–334	Hypothetical protein GQ55_7G126900 [*Panicum hallii* var. *hallii*]	9.00E‐149	FX986003
	R: GTTTCTTTACAGCACAACATAGCCCTC					
Cang_2073	F: CTATAGGGCACGCGTGGTCAGTGCAGCCGAGATTCTTG	(AAG)_10_	466–475	Putative DEAD‐box ATP‐dependent RNA helicase family protein [*Zea mays*]	0.0	FX986008
	R: GTTTCTTCCCATCTCGATCCCAAATCC					
Cang_3069	F: TGTGGAATTGTGAGCGGGTCTCCTCCGCCAAGTACTC	(AAG)_10_	398–439	Poly(A)‐specific ribonuclease PARN [*Ananas comosus*]	0.0	FX986006
	R: GTTTCTTAATTGGAGGATGGCAAAGCG					
Cang_3862	F: CACGACGTTGTAAAACGACGATCCATCCACTCTCCCTCC	(AG)_17_	173–183	Uncharacterized protein LOC100191912 [*Zea mays*]	9.00E‐113	FX986009
	R: GTTTCTTCATCCACCACGATACGCTTC					
Cang_4293	F: CTATAGGGCACGCGTGGTCGATTTCCACTGCGTGTACC	(AG)_12_	244–250	PREDICTED: WD‐40 repeat‐containing protein MSI4‐like [*Phoenix dactylifera*]	0.0	FX986001
	R: GTTTCTTCCACTCGCAAACAACAGTCG					
Cang_4398	F: CGGAGAGCCGAGAGGTGTCCTACTAAAGTCCCTGCTGAG	(AG)_12_	147–153	PREDICTED: uncharacterized protein LOC103721079	1.00E‐90	FX985994
	R: GTTTCTTGTTGGTTGAGTGAGGCTGTG					
Cang_5849	F: CACGACGTTGTAAAACGACACCCACCCATAGTTCCAGAAG	(AG)_10_	406–418	d‐cysteine desulfhydrase 2, mitochondrial‐like isoform X4	3.00E‐09	FX986007
	R: GTTTCTTACCTATGAGTCAGCCCGAAC					
Cang_7187	F: CGGAGAGCCGAGAGGTGGCAGCGTGGGAAGGAAGAG	(AG)_12_	366–409	WD repeat‐containing protein 44‐like [*Ananas comosus*]	0.0	FX986004
	R: GTTTCTTAAAGCGTTGGAAAGAGCGTC					
Cang_7240	F: CACGACGTTGTAAAACGACAAAGCTTGGCAGATTCGTCG	(AGG)_11_	210–218	PREDICTED: protein TIC 21, chloroplastic [*Musa acuminata*]	2.00E‐98	FX985998
	R: GTTTCTTAATGCAGGCGTCGATGTTAC					
Cang_7261	F: CACGACGTTGTAAAACGACCTTCGTTTCACCACAGCTGC	(AG)_12_	236–238	Protein ROOT PRIMORDIUM DEFECTIVE 1 [*Asparagus officinalis*]	0.0	FX986000
	R: GTTTCTTAAACCTCACCACTGCACTCG					
Cang_10657	F: CGGAGAGCCGAGAGGTGGAGGCGAATTGAGTTGCTCC	(AG)_12_	175–185	Probable transcription factor At5g28040 [*Ananas comosus*]	2.00E‐89	FX985996
	R: GTTTCTTGCCAATGCCAAACTTTGAGG					
Cang_18857	F: CTATAGGGCACGCGTGGTCTCTCTCAGCTCGGACAGTG	(AG)_12_	398–408	PREDICTED: protein TIFY 4B‐like isoform X4 [*Phoenix dactylifera*]	8.00E‐58	FX986005
	R: GTTTCTTTTCCACCGAAATCAGGGAGG					
Cang_19507	F: TGTGGAATTGTGAGCGGCACAGTATCTTTCTCCGCCC	(AG)_19_	201–215	PREDICTED: histone deacetylase 19‐like [*Elaeis guineensis*]	0.0	FX986010
	R: GTTTCTTAGAAGTATGAGACCCGACGC					
Cang_21384	F: CACGACGTTGTAAAACGACGGGTTACCGAGGCACAATTG	(AC)_12_	118–136	No significant similarity found.	—	FX985993
	R: GTTTCTTGATGCGACACAACTAACCCG					
Cang_22899	F: CTATAGGGCACGCGTGGTGGAGAGCAAATTCAGAGCGG	(AG)_11_	153–157	PREDICTED: transcription factor PCL1‐like [*Musa acuminata* subsp. *malaccensis*]	1.00E‐74	FX985995
	R: GTTTCTTACAGAGAGAAGCAAGGCAGG					
Cang_25819	F: CTATAGGGCACGCGTGGTGGAGTTGATGATGGGTTTAGGG	(AG)_17_	287–297	No significant similarity found.	—	FX986012
	R: GTTTCTTGGTCTGTGCCACTTAGTCCC					
Cang_46532	F: CGGAGAGCCGAGAGGTGAGCCCTAGAAACCTGACCTTG	(AC)_12_	302–305	No significant similarity found.	—	FX986002
	R: GTTTCTTGGACACTATGCTGTACAAGGG					
Cang_48335	F: TGTGGAATTGTGAGCGGAGTTGTAGGTGGTGTAGCGG	(AG)_10_	185–189	No significant similarity found.	—	FX985997
	R: GTTTCTTCCCTGGCACTGTTTAGCTTG					

**Table 2:** Characteristics of the 20 polymorphic EST‐SSR markers in three populations of *Carex angustisquama*.^a^

Locus	CA09 (*N* = 24)			CA14 (*N* = 24)			CA15 (*N* = 24)			All (*N* = 72)		
	*A*	*H*_e_	*H*_o_	*A*	*H*_e_	*H*_o_	*A*	*H*_e_	*H*_o_	*A*	*H*_e_	*H*_o_
Cang_681	1	0.000	0.000	1	0.000	0.000	3	0.544	0.238^b^	4	0.510	0.081^b^
Cang_1267	1	0.000	0.000	2	0.105	0.111	5	0.319	0.182	7	0.702	0.094^b^
Cang_1881	2	0.469	0.083^b^	1	0.000	0.000	1	0.000	0.000	3	0.525	0.030^b^
Cang_2073	1	0.000	0.000	1	0.000	0.000	2	0.466	0.217	3	0.511	0.076^b^
Cang_3069	2	0.249	0.292	1	0.000	0.000	3	0.232	0.174	5	0.703	0.167^b^
Cang_3862	1	0.000	0.000	1	0.000	0.000	1	0.000	0.000	3	0.662	0.000^b^
Cang_4293	1	0.000	0.000	1	0.000	0.000	1	0.000	0.000	3	0.663	0.000^b^
Cang_4398	1	0.000	0.000	1	0.000	0.000	1	0.000	0.000	3	0.662	0.000^b^
Cang_5849	2	0.249	0.292	1	0.000	0.000	1	0.000	0.000	2	0.100	0.106
Cang_7187	1	0.000	0.000	1	0.000	0.000	1	0.000	0.000	2	0.463	0.000^b^
Cang_7240	1	0.000	0.000	1	0.000	0.000	1	0.000	0.000	3	0.662	0.000^b^
Cang_7261	1	0.000	0.000	2	0.105	0.000	2	0.087	0.091	4	0.506	0.031^b^
Cang_10657	1	0.000	0.000	2	0.054	0.056	1	0.000	0.000	3	0.409	0.016^b^
Cang_18857	2	0.080	0.000	1	0.000	0.000	2	0.159	0.087	3	0.509	0.030^b^
Cang_19507	2	0.353	0.292	1	0.000	0.000	3	0.580	0.524	4	0.621	0.286^b^
Cang_21384	1	0.000	0.000	1	0.000	0.000	2	0.049	0.050	3	0.423	0.016^b^
Cang_22899	1	0.000	0.000	2	0.054	0.056	1	0.000	0.000	3	0.409	0.016^b^
Cang_25819	1	0.000	0.000	1	0.000	0.000	1	0.000	0.000	3	0.662	0.000^b^
Cang_46532	2	0.413	0.167^b^	1	0.000	0.000	1	0.000	0.000	3	0.517	0.061^b^
Cang_48335	2	0.041	0.042	2	0.054	0.056	3	0.275	0.188	4	0.539	0.086^b^
Average	1.350	0.093	0.058	1.55	0.019	0.014	1.8	0.136	0.088	3.4	0.538	0.055^b^

Note

*A* = number of alleles per locus; *H*_e_ = expected heterozygosity; *H*_o_ = observed heterozygosity; *N* = number of individuals genotyped.

a Voucher and locality information are provided in Appendix 1.

b Significant possibility of presence of null alleles (99% confidence level) detected by MICRO‐CHECKER (van Oosterhout et al., 2004).

**Table 3:** Cross‐amplification and genetic diversity statistics of the EST‐SSR markers developed for *Carex angustisquama* in two related species.^a^

Locus	*C. doenitzii* (*N* = 24)			*C. podogyna* (*N* = 16)		
	*A*	*H*_e_	*H*_o_	*A*	*H*_e_	*H*_o_
Cang_681	5	0.321	0.182	2	0.444	0.333
Cang_1267	4	0.574	0.292^b^	2	0.305	0.250
Cang_1881	8	0.806	0.522^b^	1	0.000	0.000
Cang_2073	6	0.747	0.87	1	0.000	0.000
Cang_3069	7	0.694	0.609	1	0.000	0.000
Cang_3862	6	0.713	0.435^b^	2	0.486	0.500
Cang_4293	5	0.712	0.783	2	0.320	0.133
Cang_4398	4	0.644	0.045^c^, ^b^	3	0.331	0.267
Cang_5849	3	0.46	0.364	1	0.000	0.000
Cang_7187	2	0.315	0.217	1	0.000	0.000
Cang_7240	6	0.753	0.375^c^, ^b^	1	0.000	0.000
Cang_7261	6	0.751	0.826	1	0.000	0.000
Cang_10657	4	0.396	0.294	2	0.444	0.000
Cang_18857	5	0.694	0.739	2	0.451	0.563
Cang_19507	7	0.794	0.762	1	0.000	0.000
Cang_21384	5	0.718	0.591	2	0.117	0.125
Cang_22899	2	0.194	0.217	1	0.000	0.000
Cang_25819	5	0.688	0.571	1	0.000	0.000
Cang_46532	2	0.258	0.217	2	0.358	0.333
Cang_48335	5	0.5	0.125^c^, ^b^	–	–	–
Average	4.85	0.587	0.452	1.450	0.163	0.125

Note

*A* = number of alleles per locus; *H*_e_ = expected heterozygosity; *H*_o_ = observed heterozygosity; *N* = number of individuals genotyped.

a Voucher and locality information are provided in Appendix 1.

b Significant possibility of presence of null alleles (99% confidence level) detected by MICRO‐CHECKER (van Oosterhout et al., 2004).

c Significant departures (*P* < 0.01) from Hardy–Weinberg equilibrium after Bonferroni correction.

EST‐SSR markers were shown to have a disadvantage of less polymorphism than genomic SSR markers (Bouck and Vision, 2007; Ellis and Burke, 2007), and we found low genetic variation in all populations of *C. angustisquama*. This may be caused by presence of null alleles. However, substantial polymorphisms were detected in *C. doenitzii*, which is the most closely related species to *C. angustisquama* (K. Nagasawa, H. Setoguchi, M. Maki, H. Goto, K. Fukushima, Y. Isagi, S. Sakaguchi, Y. Suyama, and Y. Tsunamoto, unpublished data). Moreover, in *C. angustisquama*, although most loci were homozygous within populations, these loci were fixed with different alleles for each population, which likely reflects evolutionary history rather than null alleles. Thus, we conclude that low genetic variation of *C. angustisquama* is probably caused by the species’ demographic history.

## CONCLUSIONS

The 20 EST‐SSR markers developed for *C. angustisquama* are less polymorphic within populations. However, in intraspecific and cross‐species amplification, substantial polymorphisms were detected, indicating that low genetic variation in *C. angustisquama* results from the species’ demographic history, and not from the markers’ characteristics. Thus these markers will be useful for investigating intraspecific relationships among *C. angustisquama* populations occurring in disjunct solfatara fields. These markers are also useful in other *Carex* species, providing novel population genetic tools in this speciose genus.

## DATA ACCESSIBILITY

Cleaned reads from the cDNA library have been deposited to the DNA Data Bank of Japan (DDBJ; Bioproject PRJDB6849). Sequence information for the developed primers has been deposited to the National Center for Biotechnology Information (NCBI); GenBank accession numbers are provided in Table 1.

## Appendix 1 Sample information for *Carex* species used in this study.

**Table:**

Species	Population	*N*	Collection locality	Geographic coordinates (Altitude, m)	Voucher specimen accession no.^a^
*Carex angustisquama* Franch.^b^	CA18	1	Goshogake, Senboku‐shi, Akita Pref., Japan	35°21′38″N, 137°01′34″E (1002)	KYO 00023447
*Carex angustisquama* c ^,^ d ^,^ e	CA09	24	Katanuma, Osaki‐shi, Miyagi Pref., Japan	38°44′02″N, 140°43′28″E (309)	KYO 00023438
*Carex angustisquama* d	CA13	2	Mt. Kurikoma, Ichinoseki‐shi, Iwate Pref., Japan	38°58′47″N, 140°46′10″E (1113)	KYO 00023439
*Carex angustisquama* d ^,^ e	CA14	24	Mt. Hakkoda, Aomori‐shi, Aomori Pref., Japan	40°38′56″N, 140°51′15″E (936)	KYO 00023440
*Carex angustisquama* d ^,^ e	CA15	24	Mt. Osorezan, Mutsu‐shi, Aomori Pref., Japan	41°19′47″N, 141°05′10″E (216)	KYO 00023444
*Carex doenitizii* Boeckeler^f^	C86	24	Mt. Konsei, Nikko‐shi, Gunma Pref., Japan	36°49′04″N, 139°23′37″E (2044)	KYO 00023454
*Carex podogyna* Franch. & Sav.^f^	C116	16	Mt. Shiogiri, Miyazu‐shi, Kyoto Pref., Japan	35°39′01″N, 135°12′27″E (610)	NA

## Appendix 2 Twelve monomorphic EST‐SSR markers developed for *Carex angustisquama*.

**Table:**

Locus	Primer sequences (5′–3′)	Repeat motif	Allele size range (bp)	BLASTX top hit description	*E*‐value
Cang_103	F: CACGACGTTGTAAAACGACGATCGGTGATTGGGCCTTTG	(AG)_11_	265	Glutamine synthetase root isozyme 3 [*Zea mays*]	0.0
	R: GTTTCTTGCCCTGATTTCTGAACCGTG				
Cang_594	F: CTATAGGGCACGCGTGGTTGCTCCAGTCCCAACCATAG	(AG)_20_	325	PREDICTED: calmodulin‐binding transcription activator 4 isoform X1 [*Elaeis guineensis*]	0.0
	R: GTTTCTTTGGGTGTGCTTCTGAGACC				
Cang_1002	F: TGTGGAATTGTGAGCGGCGGTGGTTGGAATTCGAAGG	(AG)_11_	434	Sulfite exporter TauE/SafE family protein 4 [*Sorghum bicolor*]	4.00E‐143
	R: GTTTCTTTCCAGTTCACCTCCAGCTTC				
Cang_1737	F: TGTGGAATTGTGAGCGGGAGAAATCAACAGAGCGGGC	(AAG)_14_	414	PREDICTED: eukaryotic translation initiation factor 3 subunit I‐like [*Phoenix dactylifera*]	0.0
	R: GTTTCTTAACTGCGATTGGTCCTGTTG				
Cang_1744	F: CACGACGTTGTAAAACGACTTCCTGGATCCTTGTCGACC	(AG)_20_	276	PREDICTED: guanine nucleotide‐binding protein‐like NSN1 [*Elaeis guineensis*]	0.0
	R: GTTTCTTGCCTACATAACCCATCGCTC				
Cang_2515	F: TGTGGAATTGTGAGCGGACCCTAGACTCGGATCCTCC	(AG)_23_	279	Carbon catabolite repressor protein 4 homolog 1‐like [*Ananas comosus*]	0.00E+00
	R: GTTTCTTGCCAGACTTATACTCTCCCTCG				
Cang_2955	F: CGGAGAGCCGAGAGGTGCTGTAACGAATCAGGTGCGG	(AAG)_10_	410	Threonine dehydratase biosynthetic, chloroplastic [*Ananas comosus*]	0.0
	R: GTTTCTTCTCCATTACCTGCTCCCTCC				
Cang_3156	F: CACGACGTTGTAAAACGACTTCAGTAGCCGAGCCTCATC	(AG)_12_	413	Eukaryotic translation initiation factor 1A [*Citrus clementina*]	5.00E‐81
	R: GTTTCTTCCTCTCTTCCTGAACAAACCG				
Cang_3166	F: CACGACGTTGTAAAACGACCGCTCTTGTGCAGTTCCAAC	(AT)_19_	206	PREDICTED: uncharacterized protein LOC107807406 isoform X1 [*Nicotiana tabacum*]	2.00E‐177
	R: GTTTCTTGGGAGAGGGATCTGAGCTTG				
Cang_3348	F: CTATAGGGCACGCGTGGTATTGCCTCCACAGCCTCC	(AG)_17_	192	PREDICTED: NADP‐dependent d‐sorbitol‐6‐phosphate dehydrogenase [*Elaeis guineensis*]	0.0
	R: GTTTCTTAGCGGATAAGAGGAGATCGC				
Cang_4013	F: TGTGGAATTGTGAGCGGACACGAAGCAGCTCTCTACC	(AG)_18_	114	Peroxisome biogenesis protein 1 [*Ananas comosus*]	0.0
	R: GTTTCTTATTCGCCTCTGAGTCGAGAC				
Cang_4089	F: CGGAGAGCCGAGAGGTGCACCTCCTCCTCTCTAAACCC	(AG)_24_	198	Auxin response factor 18 [*Ananas comosus*]	0.0
	R: GTTTCTTCTGCTCTTCTCATTGGCGTC				
